# Comparative Efficacy and Safety of Veno-Arterial Extracorporeal Membrane Oxygenation (VA-ECMO) Versus Impella for Cardiogenic Shock: A Systematic Review and Meta-Analysis

**DOI:** 10.7759/cureus.101432

**Published:** 2026-01-13

**Authors:** Khaled A Soliman, Ahmed Osman Hassan Ali, Farrukh Ameer, Mohamed Hany Elmasry, Fahd Alrumaih, Ahmad A Ibrahim, Mohammad S Ali, Hadeel J Alzahrani, Fahad M Algharbi, Abdulwahab M Albalawi, Faezah A Khaliqi

**Affiliations:** 1 Emergency Medicine, Armed Forces Hospital Southern Region, Khamis Mushait, SAU; 2 Critical Care, Dr. Soliman Fakeeh Hospital, Riyadh, SAU; 3 Cardiology, Dr. Soliman Fakeeh Hospital, Riyadh, SAU; 4 Intensive Care Unit and Anesthesia, Care Medical Hospital, Riyadh, SAU; 5 Surgery, Prince Sattam Bin Abdulaziz University, Riyadh, SAU; 6 Emergency Medicine, Riyadh Hospital, Riyadh, SAU; 7 Critical Care, Ministry of Health-Imam Abdulrahman Al Faisal Hospital, Riyadh, SAU; 8 General Medicine, Dar Aluloom University, Riyadh, SAU; 9 General Medicine, Shaqra University, Riyadh, SAU; 10 Internal Medicine, Tabuk Health Cluster, Tabuk, SAU; 11 General Practice, Arabian Gulf University, Muharraq, BHR

**Keywords:** cardiogenic shock, extracorporeal membrane oxygenation, impella, mechanical circulatory support, meta-analysis, veno-arterial extracorporeal membrane oxygenation

## Abstract

This systematic review and meta-analysis aimed to systematically evaluate the comparative efficacy and safety of Impella and veno-arterial extracorporeal membrane oxygenation (VA-ECMO) in the management of cardiogenic shock (CS). A systematic search of the Cochrane Controlled Register of Trials (CENTRAL), Medical Literature Analysis and Retrieval System Online (MEDLINE), and Scopus was conducted from inception to October 2025 to identify non-randomized and randomized studies comparing Impella and VA-ECMO in adults with CS. The review protocol was registered with the International Prospective Register of Systematic Reviews (PROSPERO) database (CRD420251158340). The primary outcome was short-term mortality. The secondary outcomes included stroke, major bleeding, and limb ischemia. The risk of bias was assessed using the Risk of Bias in Non-randomized Studies of Interventions (ROBINS-I) tool. Pooled risk ratios (RRs) with 95% confidence intervals (CIs) were calculated using a random-effects model with Knapp-Hartung adjustment. Ten non-randomized observational studies involving 5364 patients were included. The overall risk of bias was serious across all studies, primarily due to profound confounding by indication. The meta-analysis revealed no statistically significant difference in the risk of short-term mortality between patients treated with Impella and VA-ECMO (RR, 0.92; 95% CI, 0.76-1.10; p=0.30), although substantial heterogeneity was present (I² = 64.5%). In contrast, Impella use was associated with a significantly lower risk of stroke (RR, 0.52; 95% CI, 0.36-0.75), major bleeding (RR, 0.53; 95% CI, 0.49-0.57), and limb ischemia (RR, 0.55; 95% CI, 0.45-0.68). Based on observational data with a very low certainty of evidence, the use of Impella was not associated with a survival benefit compared to VA-ECMO but was associated with a more favourable safety profile. These findings are limited by confounding factors, and the choice between these devices should be individualized based on the required level of cardiorespiratory support and patient-specific risk factors. Adequately powered randomized controlled trials (RCTs) are required.

## Introduction and background

Cardiogenic shock (CS) represents a critical state of end-organ hypoperfusion stemming from primary cardiac failure and remains a formidable clinical challenge associated with mortality rates that approach 40% to 50% [1,2]. This life-threatening syndrome complicates acute myocardial infarction (AMI) but is observed in the context of advanced heart failure [3]. The pathophysiology involves a downward spiral of reduced cardiac output, systemic hypotension, and myocardial ischemia, which perpetuates further cardiac dysfunction [4]. When initial pharmacological interventions with inotropes and vasopressors prove insufficient to restore adequate perfusion, temporary mechanical circulatory support (MCS) becomes a necessary therapeutic option [3].

In the contemporary management of CS, several potent MCS devices have seen increased utilization, supplanting the routine use of intra-aortic balloon pumps (IABP) after evidence demonstrated a lack of mortality benefit [5]. Among these advanced therapies, veno-arterial extracorporeal membrane oxygenation (VA-ECMO) and percutaneous microaxial flow pumps, such as the Impella device, have emerged as two prominent strategies. However, these two modalities operate on different physiological principles. VA-ECMO provides cardiopulmonary support by draining deoxygenated venous blood, circulating it through an external oxygenator, and returning it to the arterial system, thereby ensuring systemic oxygenation and perfusion [4]. A hemodynamic consequence of retrograde aortic flow is an increase in left ventricular (LV) afterload, which can impair native cardiac recovery and precipitate pulmonary edema [1,6].

In contrast, the Impella device is a transvalvular microaxial pump that transfers blood from the left ventricle to the ascending aorta. This mechanism unloads the left ventricle, decreasing myocardial wall stress and oxygen consumption while augmenting forward cardiac output and coronary perfusion [1,7]. This fundamental difference in mechanism, LV unloading with Impella versus systemic support at the cost of increased LV afterload with VA-ECMO, has created clinical equipoise regarding the optimal initial MCS strategy for patients with CS.

Despite the widespread and increasing adoption of both devices, their comparative effectiveness remains uncertain, as the evidence base is composed almost entirely of non-randomized observational studies [1,6]. These studies are subject to a risk of confounding by indication, wherein sicker patients, often those with biventricular failure, profound metabolic derangement, or concomitant cardiac arrest, are preferentially treated with the more comprehensive support offered by VA-ECMO [7]. This selection bias complicates the interpretation of outcomes and contributes to the heterogeneous and often conflicting results in the existing literature. Consequently, definitive clinical guidance is lacking in the absence of large-scale randomized controlled trials (RCTs).

Therefore, this systematic review and meta-analysis was warranted to appraise and synthesize the existing comparative literature. By focusing on studies that have employed statistical methods to mitigate confounding, this review aims to provide a robust quantitative summary of the comparative efficacy and safety of VA-ECMO versus Impella for the management of CS, thereby informing clinical practice and identifying key knowledge gaps for future investigation.

## Review

Methods

Protocol and Registration

This systematic review and meta-analysis were conducted and reported in adherence to the Preferred Reporting Items for Systematic Reviews and Meta-Analyses (PRISMA) guidelines [8]. The review protocol was prospectively registered with the International Prospective Register of Systematic Reviews (PROSPERO) under registration number CRD420251158340.

Eligibility Criteria

Studies were included if they compared the use of VA-ECMO versus an Impella device as the primary MCS strategy in adults (≥18 years) with CS. This review considered both RCTs and non-randomized comparative studies, such as prospective or retrospective cohort studies and registry analyses. The primary outcome of interest was short-term mortality, defined as the in-hospital or 30-day mortality rate. The key secondary outcomes included major bleeding, stroke, and limb ischemia. Studies were excluded if they evaluated paediatric populations, involved concurrent initiation of VA-ECMO and Impella (e.g., ECMELLA), were case reports or small case series (<10 patients), or did not include a direct comparator group.

Information Sources and Search Strategy

A systematic search was conducted across several electronic databases, including the Cochrane Central Register of Controlled Trials (CENTRAL), Medical Literature Analysis and Retrieval System Online (MEDLINE; via PubMed), and Scopus, from inception to October 2025. The search strategy was developed with an experienced medical librarian and combined Medical Subject Headings (MeSH) and text keywords related to "cardiogenic shock," "extracorporeal membrane oxygenation," and "Impella". The search was supplemented by screening the reference lists of the included articles and relevant reviews (backward citation searching) and by searching for articles that cited the included studies (forward citation searching) to identify any additional eligible publications.

Study Selection

Following the removal of duplicate records, two investigators independently screened the titles and abstracts of all identified citations using the predefined eligibility criteria. The full-text articles of all potentially relevant studies were retrieved and assessed for final inclusion by the same two reviewers. Any disagreements throughout the screening process were resolved through discussion and consensus, or, where necessary, through adjudication by a senior investigator.

Data Extraction

Two reviewers independently extracted relevant data from the included studies using a standardized, pre-piloted data collection form. The extracted information included study characteristics (first author, publication year, and study design), patient demographics, cohort type (e.g., unadjusted and propensity score (PS)-matched), sample sizes, and outcome data for both the Impella and VA-ECMO groups. For studies reporting multiple analyses, data from the most rigorously adjusted cohort (e.g., PS-matched or inverse probability of treatment weighting (IPTW)) were preferentially extracted to minimize confounding effects. All extracted data were cross-checked for their accuracy.

Risk of Bias Assessment

The methodological quality and risk of bias for each included non-randomized study were independently evaluated by two reviewers using the Risk of Bias in Non-randomized Studies of Interventions (ROBINS-I) tool [9]. This tool assesses bias across seven domains: confounding, selection of participants, classification of interventions, deviations from intended interventions, missing data, outcome measurement, and selection of reported results. Each domain was judged to have a low, moderate, serious, or critical risk of bias, leading to an overall judgment for each study. Discrepancies in the assessments were resolved by consensus.

Data Synthesis and Statistical Analysis

All statistical analyses were performed using R software (version 4.5.1; The R Core Team, R Foundation for Statistical Computing, Vienna, Austria) with the meta and metafor packages. For dichotomous outcomes, pooled risk ratios (RRs) and corresponding 95% confidence intervals (CIs) were calculated using a random-effects model with the Knapp-Hartung adjustment to provide a more conservative estimate in the presence of heterogeneity. The Mantel-Haenszel method was used for pooling.

Statistical heterogeneity across studies was quantified using the I² statistic, with values of <25%, 25-75%, and >75% interpreted as low, moderate, and high heterogeneity, respectively. The τ² statistic was calculated to estimate the between-study variance. Publication bias for the primary outcome was assessed through visual inspection of a contour-enhanced funnel plot and tested using Egger’s linear regression test for funnel plot asymmetry, with a minimum of eight studies required for the test to be adequately powered.

To evaluate the robustness of the primary analysis, a leave-one-out sensitivity analysis was conducted, in which the meta-analysis was repeated by sequentially omitting one study at a time. Furthermore, a prespecified subgroup analysis was performed to investigate potential differences in the treatment effect between studies that used statistically adjusted cohorts (e.g., PS-matched, IPTW) and those that reported only unadjusted data. Statistical significance was set at p < 0.05 for all analyses.

Results

Study Selection

**Figure 1:**
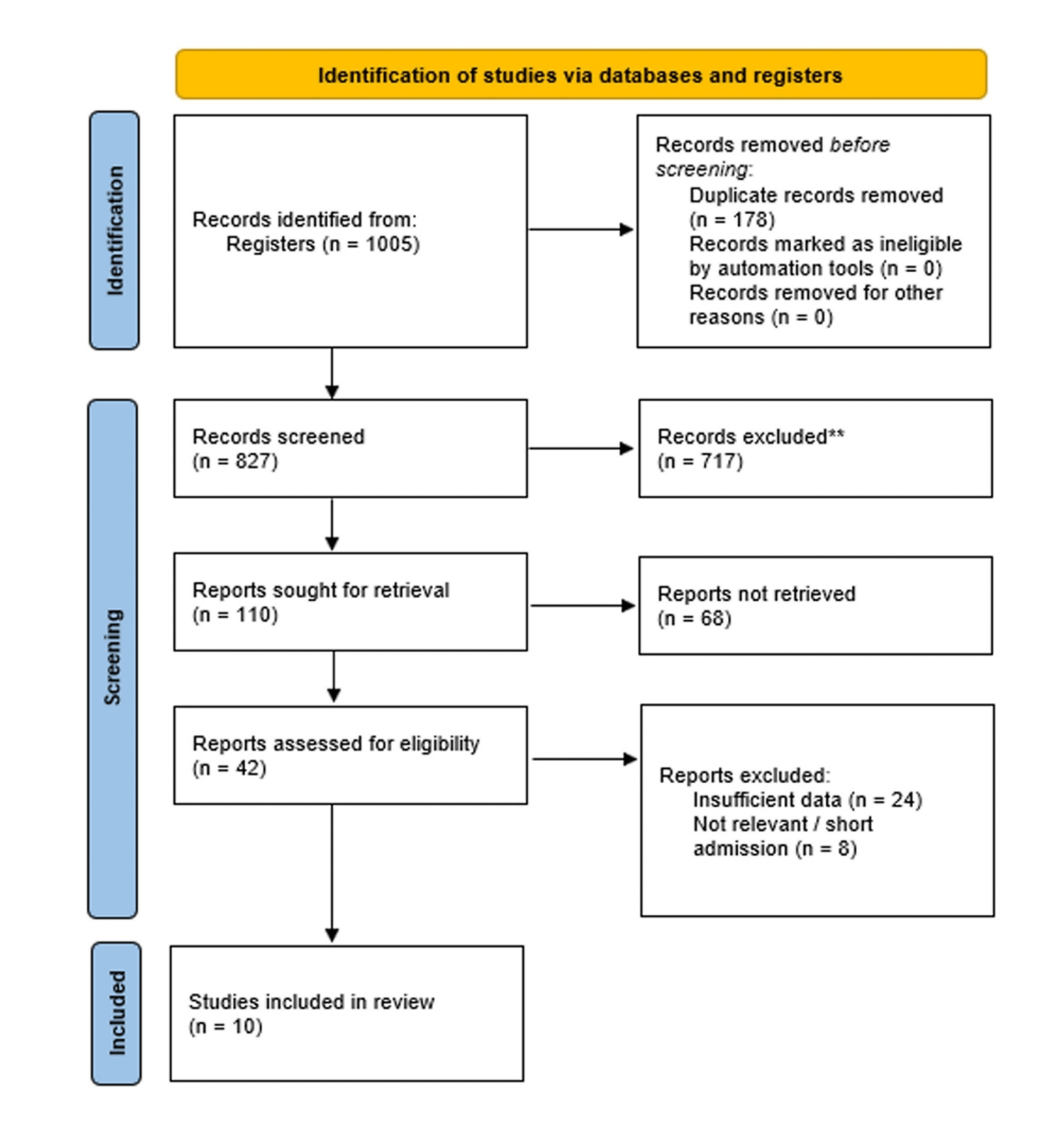
A PRISMA flowchart outlining the study selection process PRISMA: Preferred Reporting Items for Systematic Reviews and Meta-Analyses

The systematic literature search yielded 1005 records from the databases and registers. Following the removal of 178 duplicate entries, 827 unique records were screened by title and abstract, during which 717 records were excluded for not meeting the inclusion criteria. Subsequently, full-text reports of the remaining 110 articles were sought for retrieval; however, 68 of these reports could not be obtained. The full texts of the remaining 42 articles were assessed for eligibility criteria. From this group, 32 studies were excluded due to insufficient or non-extractable data (n=24) or because the patient population or intervention was not relevant (n=8). Ten comparative observational studies met the inclusion criteria and were included in the qualitative and quantitative syntheses. The detailed study selection process is illustrated in the PRISMA flowchart (Figure 1).

Characteristics of the Included Studies

The 10 included non-randomized studies were published between 2011 and 2023 and enrolled 5364 patients with CS, of whom 3546 were treated with Impella, and 1818 were treated with VA-ECMO. A methodological heterogeneity was observed across the included studies. Six studies provided statistically adjusted data using methods such as PS matching, IPTW, or multivariable adjustment [10-15]. The remaining four studies presented unadjusted comparative results [16-19]. In accordance with the pre-specified protocol, data from the most adjusted analytical cohort within each study were prioritized for extraction and inclusion in the meta-analysis to mitigate confounding biases. The primary outcome definitions varied, with some studies reporting in-hospital mortality and others reporting 30-day mortality. The detailed characteristics of each included study are summarized in Table 1.

**Table 1:** Characteristics of the included studies Abbreviations: PS, Propensity Score; IPTW, Inverse Probability of Treatment Weighting. The cohort type reflects the data prioritized for extraction in the meta-analysis.

Study	Year	Cohort Type	N (Impella)	N (ECMO)	Mortality Outcome Definition
Lamarche et al. [18]	2011	Unadjusted	29	32	30-day mortality
Mourad et al. [19]	2018	Unadjusted	19	23	30-day mortality
Garan et al. [17]	2019	Unadjusted	31	20	In-hospital mortality
Lemor et al. [10]	2020	PS Matched	450	450	In-hospital mortality
Karami et al. [15]	2020	IPTW Adjusted	90	38	30-day mortality
Karatolios et al. [11]	2021	PS Matched	83	83	In-hospital mortality
Syntila et al. [12]	2021	PS Matched	40	40	In-hospital mortality
Schurtz et al. [13]	2021	Adjusted	31	97	30-day mortality
Wernly et al. [14]	2021	PS Adjusted	73	76	30-day mortality
Bogerd et al. [16]	2023	Unadjusted	2700	959	In-hospital mortality
Total		3546	1818		

Risk of Bias Assessment

**Figure 2:**
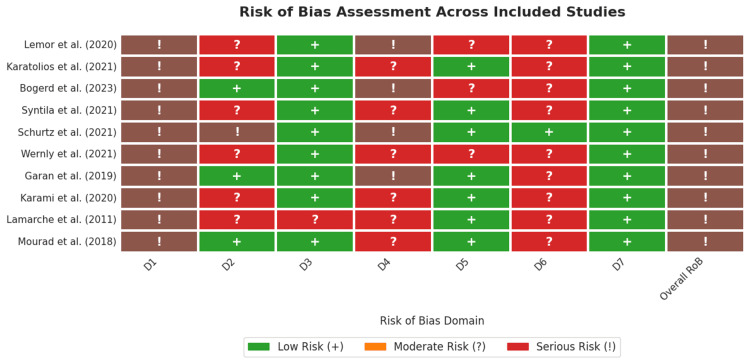
Risk of bias (RoB) assessment across the included studies A traffic light plot showing the RoB judgments for each study [10-19] across seven domains.

**Figure 3:**
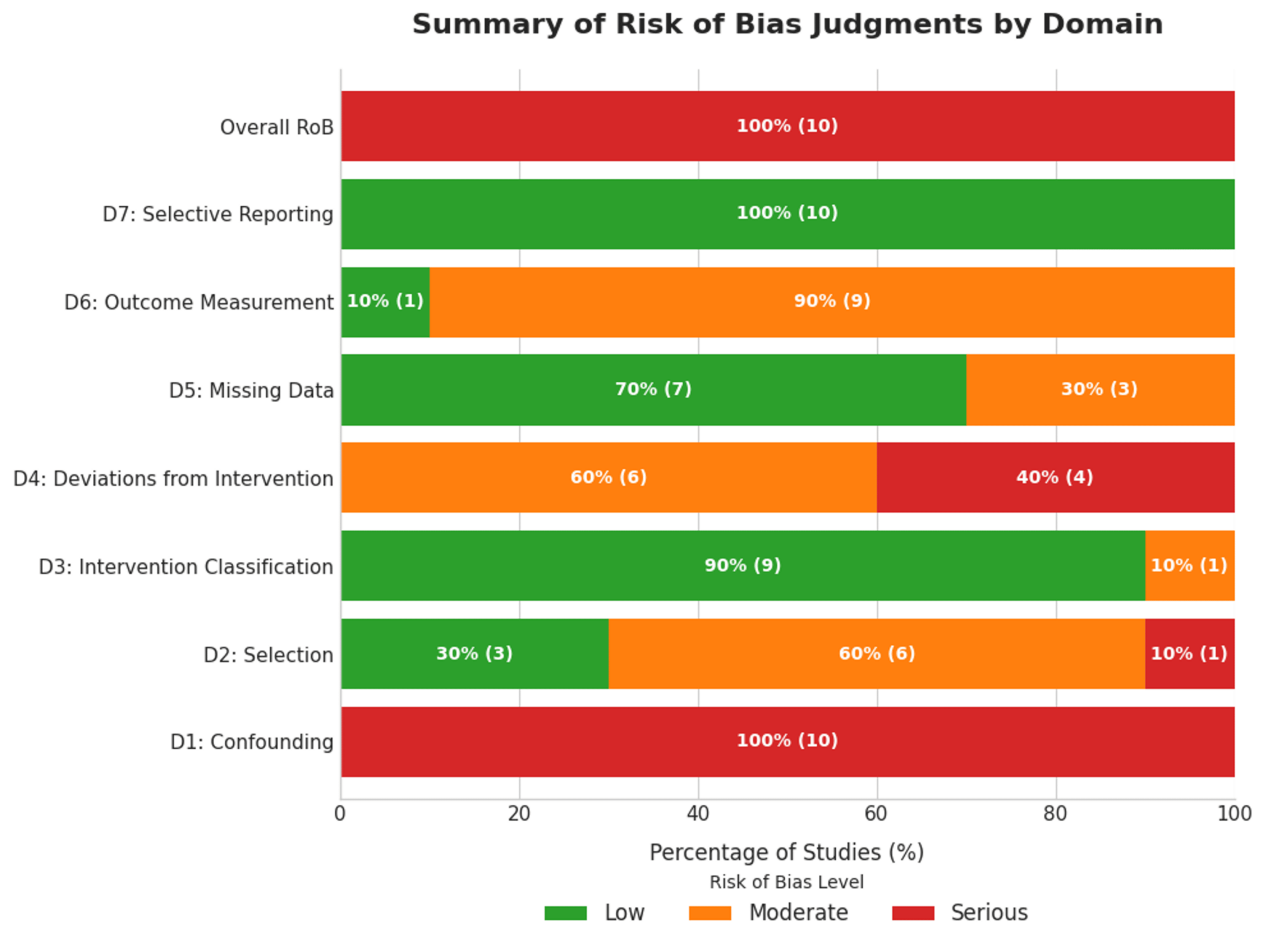
Summary of risk of bias (RoB) judgments by domain Summary bar plot presenting the percentage of studies judged at low, moderate, or serious risk of bias for each domain. The overall risk of bias was rated as serious for all included studies [10-19].

The methodological quality of the 10 included non-randomized studies was appraised using the ROBINS-I tool, with a summary of the judgments presented in Figure 2 and Figure 3. The overall risk of bias was serious across all the included studies.

A critical and universal source of bias stemmed from confounding (Domain 1), which was rated serious in 100% of the studies. This finding is inherent to the observational nature of the research question, where the initial choice of MCS device is influenced by patient acuity and clinical presentation. This confounding by indication resulted in patients with more severe hemodynamic compromise, multiorgan failure, or cardiac arrest being preferentially treated with VA-ECMO, thereby systematically biasing comparisons in favour of Impella.

Further concerns were identified in the domain of outcome measurement (Domain 6), which was judged to be at moderate risk of bias in nine of the ten studies (90%) due to the frequent use of proxy definitions or inconsistent criteria for key safety outcomes, such as defining major bleeding as the need for blood transfusion or using broad terms such as "vascular complications" without standardized adjudication. Substantial risk was also noted for deviations from the intended intervention (Domain 4), with 40% of the studies rated as serious risk and 60% as moderate risk which reflects the common clinical practice of MCS escalation (e.g. adding VA-ECMO to a patient initially treated with Impella) which constitutes a post-baseline deviation that was not adequately accounted for in the analyses of some studies.

In contrast, the risk of bias due to the classification of interventions (Domain 3) was low in 90% of the studies, indicating that the initial MCS device was well defined and reported. Moreover, there was a low risk of bias from selective reporting of results (Domain 7) across all included studies, suggesting that the authors reported all pre-specified outcomes. Despite these strengths, the pervasive and serious risk of confounding, compounded by moderate risks in outcome measurement and deviations from interventions, limits the internal validity of the included studies.

Meta-Analysis of the Outcomes

**Figure 4:**
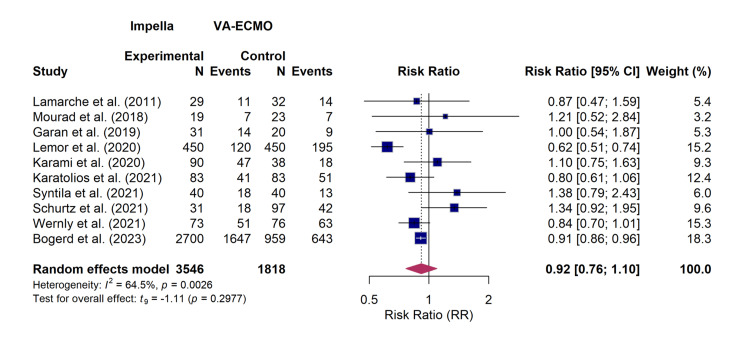
Forest plot for mortality Random-effects meta-analysis of short-term mortality comparing Impella versus veno-arterial extracorporeal membrane oxygenation (VA-ECMO). The pooled risk ratio (RR) was 0.92 (95% confidence interval (CI), 0.76–1.10), indicating no statistically significant difference in survival. The analysis included data from 10 studies: Lamarche et al. [18], Mourad et al. [19], Garan et al. [17], Lemor et al. [10], Karami et al. [15], Karatolios et al. [11], Syntila et al. [12], Schurtz et al. [13], Wernly et al. [14], and Bogerd et al. [16].

All 10 included studies, comprising 5364 patients, provided data for the primary outcome of short-term mortality. The random-effects meta-analysis did not identify a statistically significant difference in the risk of mortality between patients treated with Impella and those managed with VA-ECMO (RR, 0.92; 95% CI, 0.76-1.10; p=0.30). However, the analysis revealed substantial statistical heterogeneity among the included studies (I² = 64.5%; 95% CI, 30.1%-82.0%; p for heterogeneity = 0.0026). The forest plot detailing the individual study results and the overall pooled estimate for mortality is presented in Figure 4.

Meta-analyses of the key safety outcomes of stroke, major bleeding, and limb ischemia demonstrated statistically significant differences between the two MCS strategies.

**Figure 5:**
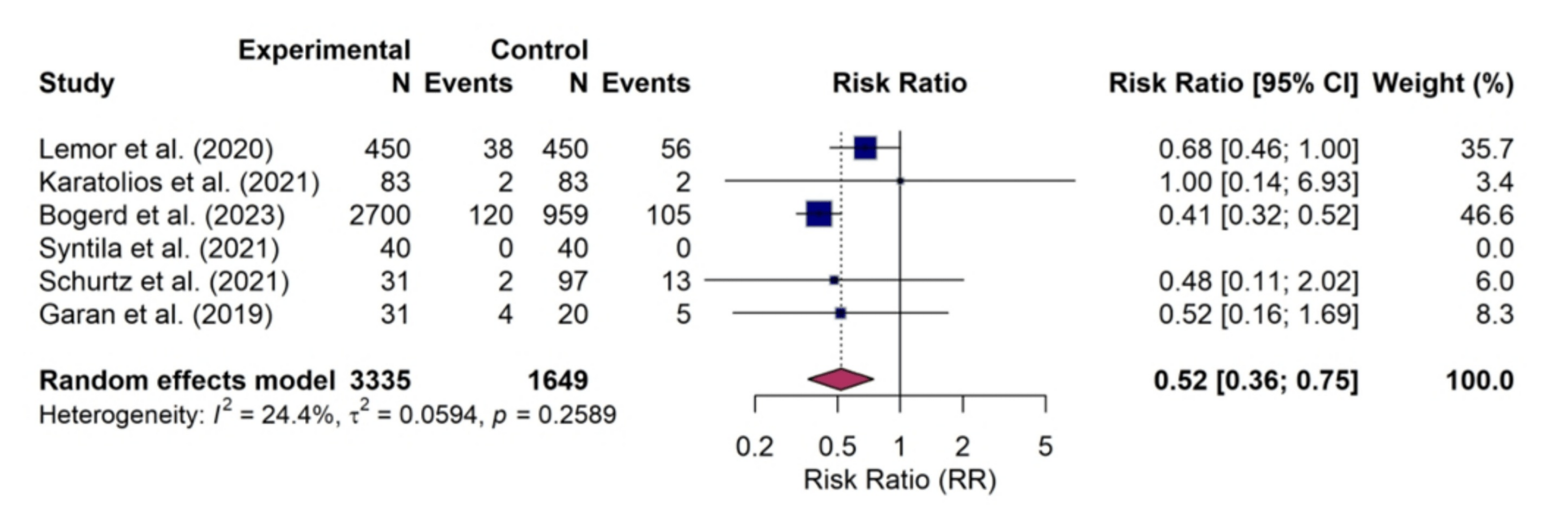
Forest plot for stroke Random-effects meta-analysis for the risk of stroke. Treatment with Impella was associated with a statistically significant reduction in stroke risk compared to veno-arterial extracorporeal membrane oxygenation (VA-ECMO) (risk ratio (RR) 0.52; 95% confidence interval (CI), 0.36–0.75). This analysis synthesized data from five studies: Lemor et al. [10], Karatolios et al. [11], Bogerd et al. [16], Schurtz et al. [13], and Garan et al. [17]. Syntila et al. [12] reported 0 events in both arms and was excluded from the pooled calculation.

Stroke: Data on the incidence of stroke were available from six studies. One study [12] reported 0 events in both treatment arms and was therefore excluded from the quantitative synthesis, leaving five studies for meta-analysis. The pooled analysis demonstrated a statistically significant reduction in the risk of stroke associated with the use of Impella compared to VA-ECMO (RR, 0.52; 95% CI, 0.36-0.75; p=0.007). Statistical heterogeneity for this outcome was low (I² = 24.4%). The corresponding forest plot is shown in Figure 5.

**Figure 6:**
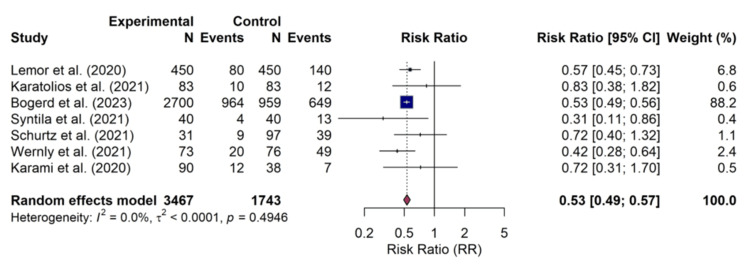
Forest plot of major bleeding Random-effects meta-analysis for the risk of major bleeding. Impella use was associated with a significantly lower risk of major bleeding compared to veno-arterial extracorporeal membrane oxygenation (VA-ECMO) (risk ratio (RR) 0.53; 95% confidence interval (CI), 0.49–0.57) with no observed heterogeneity (I^2^=0.0%I2=0.0%). The analysis included seven studies: Lemor et al. [10], Karatolios et al. [11], Bogerd et al. [16], Syntila et al. [12], Schurtz et al. [13], Wernly et al. [14], and Karami et al. [15].

Major bleeding: Seven studies reported outcomes for major bleeding. It should be noted that the definitions for this outcome varied across studies and were based on proxies such as the requirement for blood transfusion. The meta-analysis indicated that treatment with Impella was associated with a significantly lower risk of major bleeding than VA-ECMO (RR, 0.53; 95% CI, 0.49-0.57; p<0.001). No statistical heterogeneity was detected among the contributing studies (I² = 0.0%), suggesting a consistent effect across the literature. The forest plot of major bleeding is shown in Figure 6.

**Figure 7:**
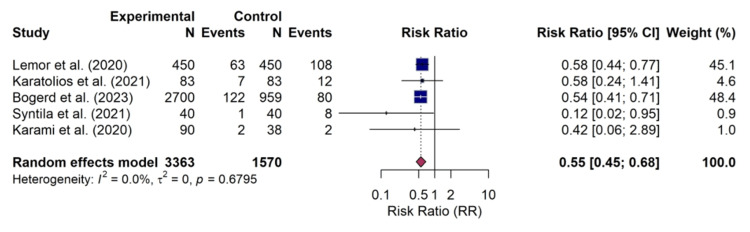
Forest plot for limb ischemia Random-effects meta-analysis for the risk of limb ischemia. The use of Impella was associated with a significantly reduced risk compared to veno-arterial extracorporeal membrane oxygenation (VA-ECMO) (risk ratio (RR) 0.55; 95% confidence interval (CI), 0.45–0.68). This outcome was reported in five studies: Lemor et al. [10], Karatolios et al. [11], Bogerd et al. [16], Syntila et al. [12], and Karami et al. [15].

Limb ischemia: Five studies provided sufficient data for inclusion in the meta-analysis for limb ischemia. The pooled results showed that the use of Impella was associated with a significantly reduced risk of limb ischemia compared to VA-ECMO (RR, 0.55; 95% CI, 0.45-0.68; p=0.001). Consistent with the findings for major bleeding, no statistical heterogeneity was identified among the studies (I² = 0.0%). The forest plot of this analysis is shown in Figure 7.

Assessment of Reporting Bias

**Figure 8:**
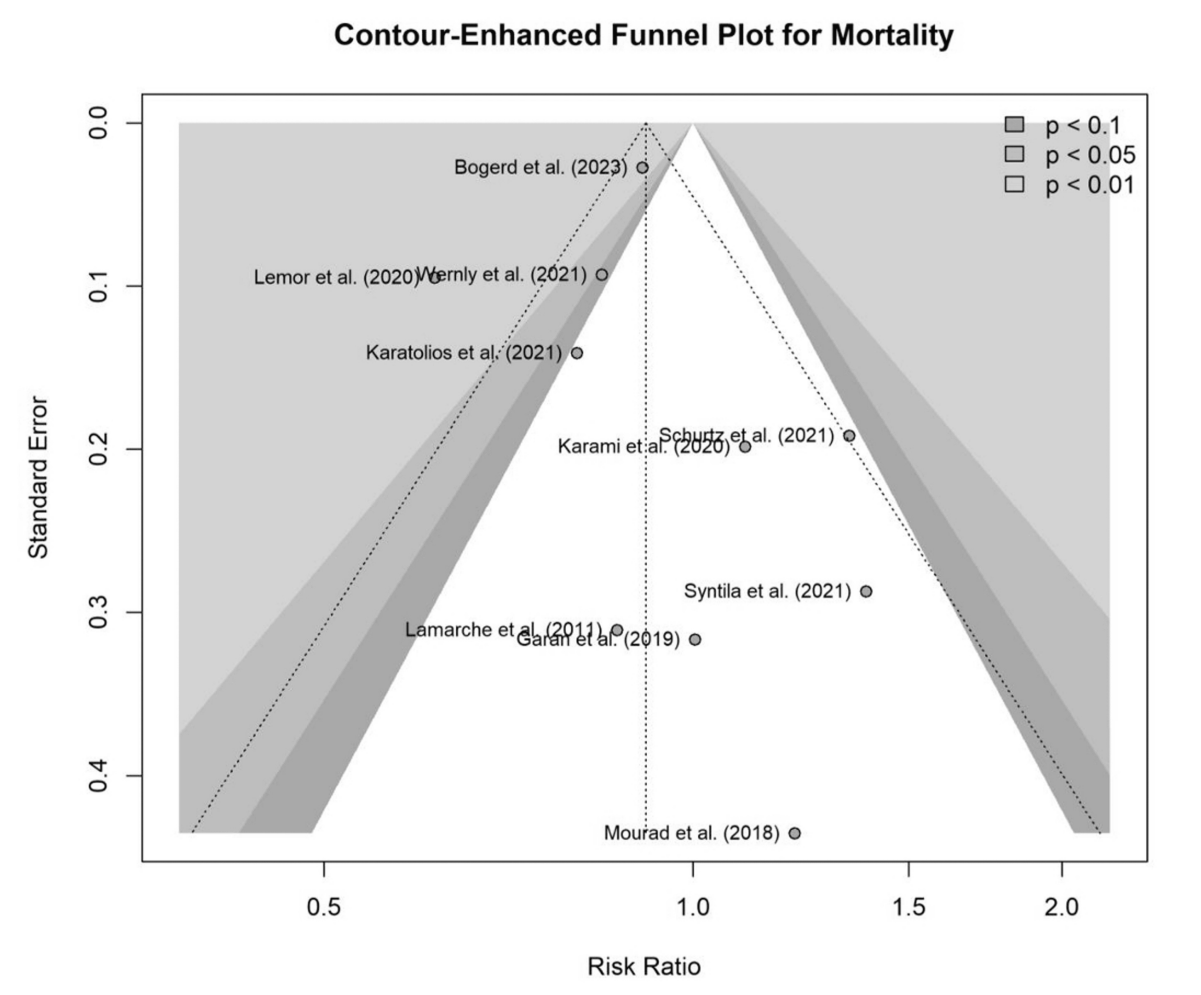
Funnel plot for mortality Contour-enhanced funnel plot assessing potential publication bias for the primary outcome of short-term mortality. The symmetrical distribution of the 10 included studies around the pooled effect estimate suggests a low likelihood of publication bias [10-19].

The potential for reporting bias in the primary outcome of short-term mortality was evaluated. Visual inspection of the contour-enhanced funnel plot (Figure 8) revealed a symmetrical distribution of the 10 included studies around the pooled effect estimate, suggesting a low likelihood of a publication bias. This qualitative assessment was supported by formal statistical tests. Egger’s linear regression test for funnel plot asymmetry was non-significant (t = 0.31, df = 8, p=0.76), providing no statistical evidence of small-study effects.

Additional Analyses

To assess the robustness of the primary outcome and explore the substantial heterogeneity observed, both a leave-one-out sensitivity analysis and a pre-specified subgroup analysis were performed.

**Figure 9:**
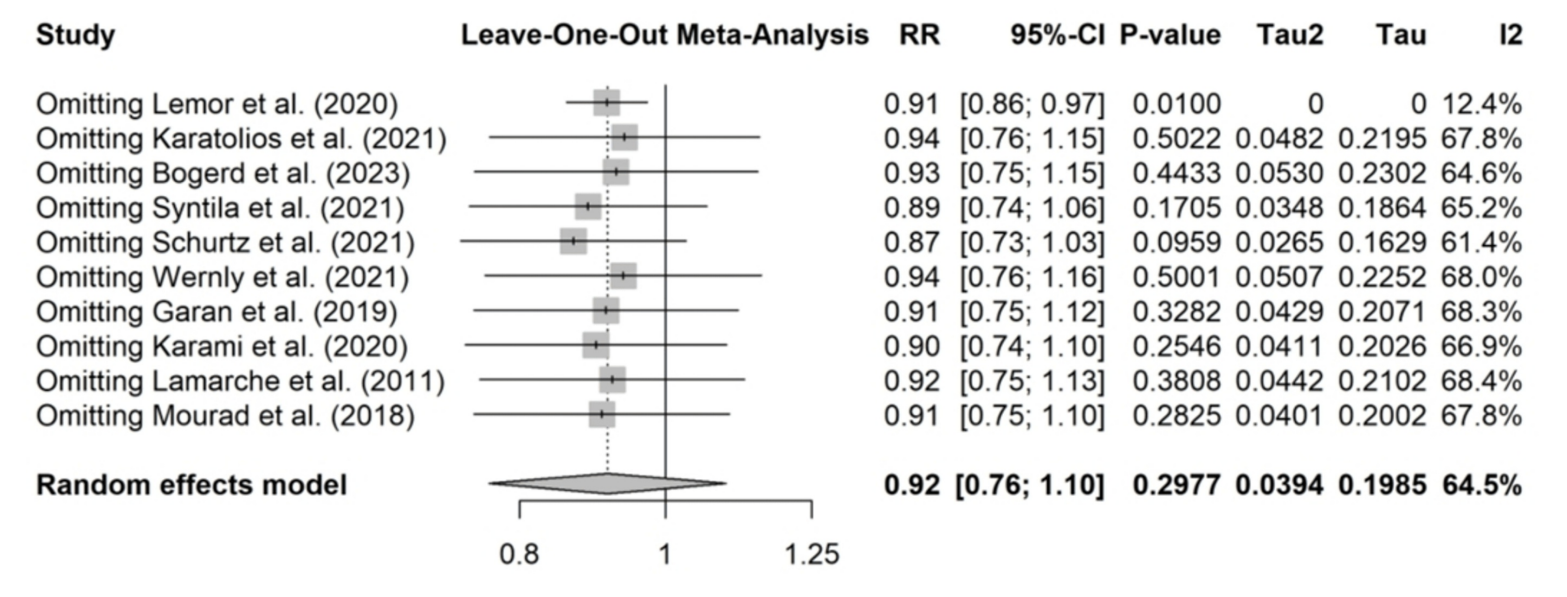
Leave-one-out meta-analysis Sensitivity analysis for short-term mortality. The forest plot displays the recalculated pooled risk ratios (RR) and heterogeneity (I^2^) when sequentially omitting each study. The omission of any single study did not result in a statistically significant change to the overall finding, demonstrating the stability of the primary result.

A sensitivity analysis was conducted for the primary outcome of mortality by removing one study at a time and recalculating the pooled effect estimates. The results presented in Figure 9 demonstrate that the omission of any single study did not significantly alter the overall pooled RR or its 95% CI. The summary estimate remained non-significant in all iterations, indicating that the overall finding for mortality was not driven by any individual study.

A subgroup analysis was conducted to investigate the influence of statistical adjustment on the pooled mortality estimate. The studies were stratified into two groups: those providing data from adjusted cohorts (e.g., PS-matched or inverse probability of treatment-weighted) and those providing only unadjusted data.

**Figure 10:**
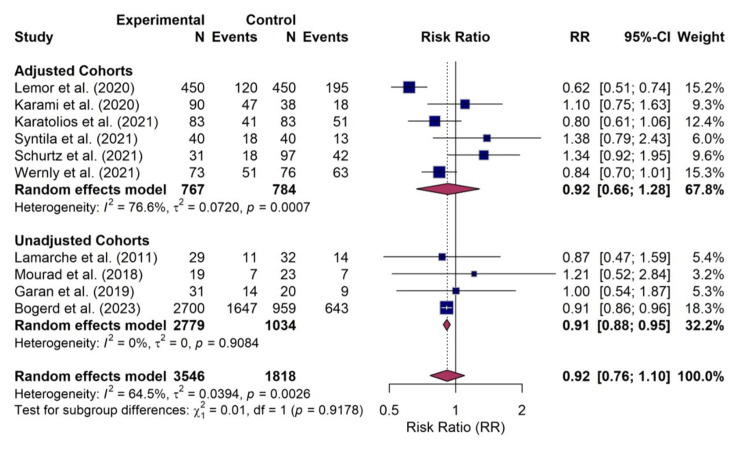
Subgroup analysis forest plot Meta-analysis of short-term mortality stratified by cohort adjustment methodology. The "Adjusted Cohorts" subgroup includes six studies [10-15] using propensity score matching or inverse probability of treatment weighting. The "Unadjusted Cohorts" subgroup includes four studies [16-19]. The test for subgroup differences was not significant (p=0.92p=0.92).

The analysis of the six adjusted cohorts (n=767 Impella, n=784 ECMO) showed a pooled RR of 0.92 (95% CI, 0.66-1.28) with considerable heterogeneity (I² = 76.6%). In contrast, the analysis of the four unadjusted cohorts (n=2779 Impella, n=1034 ECMO) yielded a pooled risk ratio of 0.91 (95% CI, 0.86-0.95), with no heterogeneity (I² = 0%). The test for subgroup differences between the adjusted and unadjusted cohorts was not statistically significant (p=0.92), suggesting that the method of adjustment did not formally modify the treatment effect in this study. The forest plot of this subgroup analysis is presented in Figure 10.

Discussion

Principal Findings

This systematic review and meta-analysis synthesized data from 10 comparative observational studies involving 5364 patients to evaluate the efficacy and safety of Impella versus VA-ECMO in CS. The primary finding of this analysis was that there was no statistically significant difference in the risk of short-term mortality between patients treated with Impella and those who received VA-ECMO. In contrast, the analysis of secondary safety outcomes demonstrated that the use of Impella was associated with a statistically significant reduction in the risks of stroke, major bleeding, and limb ischemia. However, these findings must be interpreted in the context of the substantial limitations of the underlying evidence.

Interpretation of Findings and Limitations of the Evidence

The most critical limitation of this meta-analysis is the very low certainty of the synthesized evidence, which stems from the serious risk of bias inherent in the included non-randomized studies. As highlighted by the ROBINS-I assessment, all 10 studies were judged to be at an overall serious risk of bias due to profound and unresolvable confounding factors. This form of selection bias arises because the clinical decision to use VA-ECMO is reserved for patients with more severe illnesses, such as biventricular failure, concomitant respiratory collapse, post-cardiac arrest shock, or profound metabolic derangement [3]. Consequently, patients in the VA-ECMO arms of these studies had a higher baseline risk of both mortality and complications, which systematically biased the results in favour of Impella. Although several included studies attempted to mitigate this through statistical adjustment methods, such as PS matching, these techniques can only account for measured confounders and are unlikely to fully resolve bias from unmeasured factors related to clinical acuity [10,14].

This bias explains the contradictory findings of the meta-analysis: a neutral mortality result despite a better safety profile for the Impella. The higher rates of stroke, major bleeding, and limb ischemia observed in the VA-ECMO group may be partially attributable to the device's characteristics, such as the need for large-bore cannulas and non-physiologic retrograde flow; however, they are also likely markers of a sicker patient population that is more susceptible to such complications. The substantial statistical heterogeneity (I²=64.5%) observed for the mortality outcome further supports this notion of underlying clinical and methodological diversity that could not be fully addressed in this study. The leave-one-out sensitivity analysis confirmed that no single study disproportionately influenced this primary finding; rather, heterogeneity is a systemic issue across the body of evidence. Furthermore, inconsistent and often non-standardized proxy definitions for outcomes, such as major bleeding, as noted during data extraction, introduce measurement bias and further temper confidence in these secondary findings. Therefore, while the results suggest a superior safety profile for Impella, this conclusion cannot be reliably disentangled from the underlying confounding factors.

Comparison with the Existing Literature

The findings of this meta-analysis are consistent with those of previously published systematic reviews. For instance, the review by Batchelor et al. [1], which focused specifically on CS complicating AMI and included six observational studies, reported a reduction in in-hospital mortality with Impella, which was attenuated in propensity-matched analyses. Similarly, a more recent meta-analysis by Ahmad et al. [6], involving 13 studies, found that Impella use was associated with lower in-hospital mortality and fewer device-related complications than ECMO. The present analysis builds upon and updates this evidence by including a comprehensive set of 10 recent studies, prioritizing the most rigorously adjusted data from each study, and conducting formal subgroup and sensitivity analyses to rigorously probe the data. The consistent conclusion across these reviews is that while unadjusted data often favour Impella, the effect diminishes when accounting for confounding, and no definitive survival advantage for either device can be established from the existing observational studies.

The findings of this meta-analysis must be contextualized within the rapid evolution of MCS, as our observation of a neutral mortality benefit, but superior safety profile for Impella contrasts slightly with the recent meta-analysis by Stub et al. [7] as in their review of five PS-matched studies, they reported a significant survival advantage for Impella (OR 0.57) alongside a reduction in bleeding events. While our analysis also trended toward a survival benefit for Impella, the inclusion of a broader range of studies and recent large-scale registry data, such as Bogerd et al. [16], contributed to the non-significant statistical result, reflecting the heterogeneity of the broader shock population.

However, our safety findings are robustly supported by high-quality evidence from the randomized domain as the ECLS-SHOCK ((Extracorporeal Life Support in Infarct-Related Cardiogenic Shock) trial [20], which compared VA-ECMO to medical therapy in infarct-related CS, found no reduction in 30-day mortality with ECMO but observed a significant increase in moderate or severe bleeding (23.4% vs. 9.6%) and peripheral vascular complications. These RCT results validate the safety signals detected in our meta-analysis, confirming that the large-bore cannulation and anticoagulation requirements of VA-ECMO carry intrinsic risks that are significantly higher than those of the microaxial Impella platform.

Clinical and Pathophysiological Implications

Although this meta-analysis does not support the superiority of one device over the other for survival, the consistent safety signals have important clinical implications that align with the distinct physiological mechanisms of each device used. The lower rates of limb ischemia and major bleeding with Impella are attributable to the smaller arterial access sheath required compared to the large-bore cannulas necessary for VA-ECMO [15]. Likewise, the reduced risk of stroke with Impella may be related to the antegrade and more physiological flow it generates, which contrasts with the static blood column and increased LV afterload created by the retrograde flow from VA-ECMO, both of which can promote LV thrombus formation [10].

These findings suggest that the clinical choice between Impella and VA-ECMO should not be framed as a question of universal superiority but rather as a patient-specific decision that involves a trade-off between the level of support required and the associated risk profiles. In patients with isolated predominant left ventricular (LV) failure without severe respiratory compromise or profound circulatory collapse (e.g., Society for Cardiovascular Angiography and Interventions (SCAI) Stage C or early stage D), direct LV unloading and a more favourable safety profile of Impella may represent an optimal initial strategy [4]. Conversely, in patients with biventricular failure, concomitant severe respiratory failure, refractory cardiac arrest, or profound shock with multiorgan failure (e.g., SCAI Stage E), the comprehensive biventricular and respiratory support afforded by VA-ECMO remains indispensable despite its higher complication risk [2].

The divergent safety profiles observed can be attributed to device-specific mechanics and access requirements. As highlighted by Geppert et al. [21], the Impella device operates on the principle of direct LV unloading, which reduces wall stress and oxygen consumption while augmenting forward flow, which contrasts with the retrograde aortic flow of VA-ECMO, which increases LV afterload and may necessitate higher anticoagulation targets, thereby increasing bleeding risk. Geppert et al. [21] further note that while initial studies showed no mortality benefit for Impella, major adverse cardiac and cerebrovascular event (MACCE) rates have declined over the last decade due to improved operator experience and technical refinements, a trend that may explain the favorable safety signals seen in the more recent observational cohorts included in our review.

Strengths of This Review

The primary strength of this review lies in its rigorous methodology. The protocol was prospectively registered, and the search strategy was comprehensive. By adhering to the PRISMA guidelines and employing the robust ROBINS-I tool, we provided a critical appraisal of the quality of the available evidence. A key methodological strength was the a priori decision to extract data from the most statistically adjusted cohorts available, thereby presenting a pooled estimate that best mitigates, although does not eliminate, pervasive confounding. Finally, the inclusion of multiple additional analyses, including leave-one-out sensitivity and subgroup analyses, enhanced the robustness and credibility of our findings.

Implications for Future Research

The profound limitations and serious risk of bias identified across all included studies underscore the urgent and unequivocal need for adequately powered multicenter RCTs to compare Impella and VA-ECMO. Clinical equipoise is clear, but such trials must be designed to succeed. Future trials should incorporate standardized, objective risk stratification tools at enrolment, such as the SCAI shock classification, to ensure that comparable patient populations are randomized [3]. Furthermore, the adoption of consensus-defined core outcome sets, particularly for bleeding and vascular complications, is essential to avoid the measurement heterogeneity that plagues the current literature. While awaiting evidence from RCTs, prospective multicenter registries that capture granular hemodynamic data and adhere to standardized definitions can provide higher-quality observational evidence to guide clinical decision-making.

## Conclusions

This systematic review and meta-analysis of observational studies found no significant difference in short-term mortality between patients with cardiogenic shock treated with Impella and those treated with VA-ECMO. However, Impella use was associated with a significantly lower risk of stroke, major bleeding, and limb ischemia. These findings are based on evidence of very low certainty and must be interpreted with considerable caution, as they are constrained by the serious risk of confounding by indication inherent to the non-randomized design of all the included studies. Consequently, the choice between these two MCS modalities should be individualized, weighing the comprehensive cardiorespiratory support of VA-ECMO against the more favourable safety profile of Impella based on the specific clinical and hemodynamic profile of the patient. Definitive evidence to guide the selection of the optimal initial MCS strategy in this high-risk population awaits completion of well-designed, adequately powered randomized controlled trials.

## References

[1] Batchelor RJ, Wheelahan A, Zheng WC, Stub D, Yang Y, Chan W (2022). Impella versus venoarterial extracorporeal membrane oxygenation for acute myocardial infarction cardiogenic shock: a systematic review and meta-analysis. J Clin Med.

[2] Møller JE, Hassager C, Proudfoot A (2025). Cardiogenic shock: diagnosis, phenotyping and management. Intensive Care Med.

[3] Sarma D, Jentzer JC (2024). Cardiogenic shock: pathogenesis, classification, and management. Crit Care Clin.

[4] Jumean M, Kar B (2024). Cardiogenic shock. Management of Acute and Chronic Severe Heart Failure.

[5] Murugiah K, McDonagh TA, Cohen DJ, Dhruva SS (2025). Mechanical circulatory support in acute myocardial infarction-cardiogenic shock: 2025 acute coronary syndrome guideline in context. J Am Coll Cardiol.

[6] Ahmad S, Ahsan MJ, Ikram S (2023). Impella versus extracorporeal membranous oxygenation (ECMO) for cardiogenic shock: a systematic review and meta-analysis. Curr Probl Cardiol.

[7] Stub D, Chan W, Ball J (2025). Impella compared to venoarterial extracorporeal membrane oxygenation in cardiogenic shock: a systematic review and meta-analysis of propensity score-matched studies. Shock.

[8] Moher D, Liberati A, Tetzlaff J, Altman DG (2009). Preferred Reporting Items for Systematic Reviews and Meta-Analyses: the PRISMA statement. PLoS Med.

[9] Sterne JA, Hernán MA, Reeves BC (2016). ROBINS-I: a tool for assessing risk of bias in non-randomised studies of interventions. BMJ.

[10] Lemor A, Hosseini Dehkordi SH, Basir MB (2020). Impella versus extracorporeal membrane oxygenation for acute myocardial infarction cardiogenic shock. Cardiovasc Revasc Med.

[11] Karatolios K, Chatzis G, Markus B (2021). Comparison of mechanical circulatory support with venoarterial extracorporeal membrane oxygenation or Impella for patients with cardiogenic shock: a propensity-matched analysis. Clin Res Cardiol.

[12] Syntila S, Chatzis G, Markus B (2021). Comparison of mechanical support with impella or extracorporeal life support in post-cardiac arrest cardiogenic shock: a propensity scoring matching analysis. J Clin Med.

[13] Schurtz G, Rousse N, Saura O (2021). IMPELLA(®) or extracorporeal membrane oxygenation for left ventricular dominant refractory cardiogenic shock. J Clin Med.

[14] Wernly B, Karami M, Engström AE (2021). Impella versus extracorporal life support in cardiogenic shock: a propensity score adjusted analysis. ESC Heart Fail.

[15] Karami M, den Uil CA, Ouweneel DM (2020). Mechanical circulatory support in cardiogenic shock from acute myocardial infarction: Impella CP/5.0 versus ECMO. Eur Heart J Acute Cardiovasc Care.

[16] Bogerd M, Ten Berg S, Peters EJ (2023). Impella and venoarterial extracorporeal membrane oxygenation in cardiogenic shock complicating acute myocardial infarction. Eur J Heart Fail.

[17] Garan AR, Takeda K, Salna M (2019). Prospective comparison of a percutaneous ventricular assist device and venoarterial extracorporeal membrane oxygenation for patients with cardiogenic shock following acute myocardial infarction. J Am Heart Assoc.

[18] Lamarche Y, Cheung A, Ignaszewski A, Higgins J, Kaan A, Griesdale DE, Moss R (2011). Comparative outcomes in cardiogenic shock patients managed with Impella microaxial pump or extracorporeal life support. J Thorac Cardiovasc Surg.

[19] Mourad M, Gaudard P, De La Arena P (2018). Circulatory support with extracorporeal membrane oxygenation and/or Impella for cardiogenic shock during myocardial infarction. ASAIO J.

[20] Thiele H, Zeymer U, Akin I (2023). Extracorporeal life support in infarct-related cardiogenic shock. N Engl J Med.

[21] Geppert A, Mashayekhi K, Huber K (2024). The use of mechanical circulatory support in elective high-risk percutaneous coronary interventions: a literature-based review. Eur Heart J Open.

